# Supplementary material for: Identification of photoperiod-induced specific miRNAs in the adrenal glands of Sunite sheep (Ovis aries)
Source: Front Vet Sci. 2022 Jul 22;9:888207. doi: 10.3389/fvets.2022.888207 (PMC9354845; doi:10.3389/fvets.2022.888207)
Supplement: Supplementary file 1 [file Data_Sheet_1.ZIP › Supplementary file/miRDeep2 file.zip!/miRDeep2 file/Know_Structure/SP42A2/oar-mir-411a.pdf]

miRBase precursor : oar-mir-411a
Total read count : 47647
oar-miR-411a-5p read count : 43473
oar-miR-411a-3p read count : 4165
remaining reads : 9

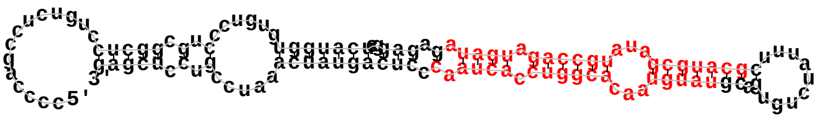

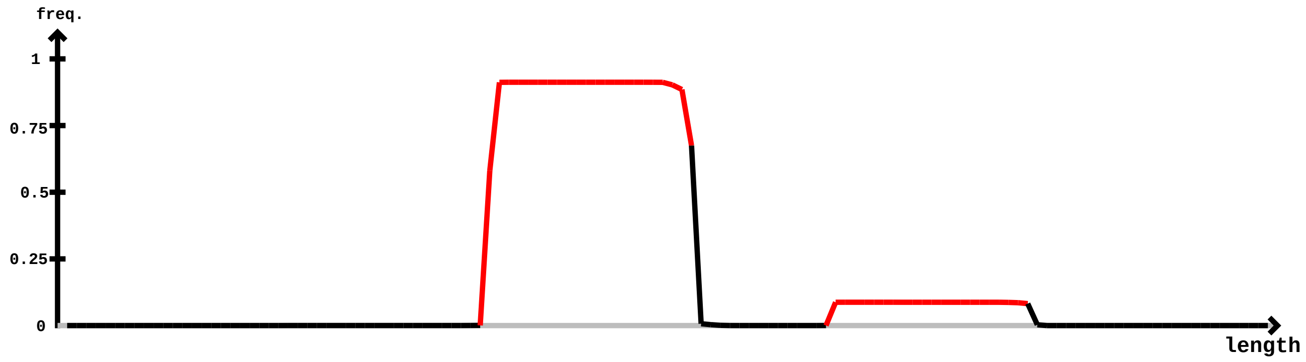


```
                                                   oar-miR-411a-3p
                                          oar-miR-411a-5p
5'- ccccagccucuguccucggcgucccugugugguacucggagagauaguagaccguauagcguacgcuuuaucugugacguauguaacacgguccacuaacccucaguaucaaauccguccucgag   -3'   exp
    ..............(((((.(.(......(((((((..(((.(.((((.((((((...((((((((.........).)))))))...)))))).)))).).))))))))))......).).)))))   reads  mm
    .....................ucccugugugguacucggagaga.................................................................................   1      0
    ......................cccugugugguacucggagaga.................................................................................   3      0
    .......................ccugugugguacucggagag..................................................................................   1      0
    ........................cugugugguacucggagag..................................................................................   1      0
    ........................cugugugguacucggagagau................................................................................   1      0
    .........................................agauaguagaccguauagcg................................................................   2      0
    .........................................agauaguagaccguauagcgu...............................................................   4      0
    .........................................agauaguagaccguauagcgua..............................................................   1      0
    .........................................agauaguagaccguauagcguacg............................................................   1      0
    ..........................................gauaguagaccguauagcgu...............................................................   3      0
    ..........................................gauaguagaccguauagcguac.............................................................   4      0
    ..........................................gauaguagaccguauagcguacg............................................................   8      0
    ...........................................auaguagaccguauagc.................................................................   3      0
    ...........................................auaguagaccguauagcg................................................................   8      0
    ...........................................auaguagaccguauagcgu...............................................................   388    0
    ...........................................auaguagaccguauagcgua..............................................................   605    0
    ...........................................auaguagaccguauagcguac.............................................................   6149   0
    ...........................................auaguagaccguauagcguacg............................................................   20396  0
    ...........................................auaguagaccguauagcguacgc...........................................................   75     0
    ...........................................auaguagaccguauagcguacgcu..........................................................   36     0
    ...........................................auaguagaccguauagcguacgcuu.........................................................   1      0
    ............................................uaguagaccguauagcg................................................................   1      0
    ............................................uaguagaccguauagcgu...............................................................   60     0
    ............................................uaguagaccguauagcgua..............................................................   224    0
    ............................................uaguagaccguauagcguac.............................................................   3865   0
    ............................................uaguagaccguauagcguacg............................................................   11410  0
    ............................................uaguagaccguauagcguacgc...........................................................   96     0
    ............................................uaguagaccguauagcguacgcu..........................................................   74     0
    ............................................uaguagaccguauagcguacgcuu.........................................................   36     0
    ............................................uaguagaccguauagcguacgcuuu........................................................   4      0
    .............................................aguagaccguauagcguac.............................................................   3      0
    .............................................aguagaccguauagcguacg............................................................   7      0
    .............................................aguagaccguauagcguacgcu..........................................................   7      0
    .............................................aguagaccguauagcguacgcuuu........................................................   1      0
```

```
                                                                                oar-miR-411a-3p
                                           oar-miR-411a-5p
ccccagccucuguccucggcgucccugugugguacucggagagauaguagaccguauagcguacgcuuuaucugugacguauguaacacgguccacuaacccucaguaucaaauccguccucgag

..............................................guagaccguauagcguacg............................................................    1    0
.................................................................cuuuaucugugacguauguaa.......................................    1    0
..........................................................................ugacguauguaacacgguccacu............................    1    0
..............................................................................guauguaacacgguccacu............................    4    0
..............................................................................guauguaacacgguccacua...........................    1    0
..............................................................................guauguaacacgguccacuaac.........................    1    0
..............................................................................guauguaacacgguccacuaacc........................    1    0
...............................................................................uauguaacacgguccacu............................    26   0
...............................................................................uauguaacacgguccacua...........................    52   0
...............................................................................uauguaacacgguccacuaa..........................    158  0
...............................................................................uauguaacacgguccacuaac.........................    3778 0
...............................................................................uauguaacacgguccacuaacc........................    141  0
................................................................................auguaacacgguccacuaa..........................    1    0
................................................................................auguaacacgguccacuaac.........................    2    0
```
